# Evaluation of an alternative ruxolitinib dosing regimen in patients with myelofibrosis: an open-label phase 2 study

**DOI:** 10.1186/s13045-018-0642-0

**Published:** 2018-08-07

**Authors:** Moshe Talpaz, Susan Erickson-Viitanen, Kevin Hou, Solomon Hamburg, Maria R. Baer

**Affiliations:** 1 University of Michigan, Ann Arbor, MI USA; 2 Incyte Corporation, Wilmington, DE USA; 3 Tower Hematology Oncology Medical Group, Beverly Hills, CA USA; 4 University of Maryland, Greenebaum Comprehensive Cancer Center, 22 S. Greene St, Baltimore, MD 21201 USA

**Keywords:** Anemia, Janus kinase, Myelofibrosis, Ruxolitinib, Thrombocytopenia, Transfusion

## Abstract

**Background:**

Ruxolitinib improves splenomegaly and symptoms in patients with intermediate-2 or high-risk myelofibrosis; however, nearly half develop grade 3/4 anemia and/or thrombocytopenia, necessitating dose reductions and/or transfusions. We report findings from an open-label phase 2 study exploring a dose-escalation strategy aimed at preserving clinical benefit while reducing hematological adverse events early in ruxolitinib treatment.

**Methods:**

Patients with myelofibrosis received ruxolitinib 10 mg twice daily (BID), with incremental increases of 5 mg BID at weeks 12 and 18 for lack of efficacy (maximum, 20 mg BID). Symptom severity was measured using the Myelofibrosis Symptom Assessment Form Total Symptom Score (MFSAF TSS).

**Results:**

Forty-five patients were enrolled, 68.9% of whom had a Dynamic International Prognostic Scoring System score of 1 to 2 (i.e., intermediate-1 disease risk). Median percentage change in spleen volume from baseline to week 24 was − 17.3% (≥ 10% reduction achieved by 26 patients [57.8%]), with a clear dose response. Median percentage change in MFSAF TSS from baseline at week 24 was − 45.6%, also with a dose response. The most frequent treatment-emergent adverse events were anemia (26.7%), fatigue (22.2%), and arthralgias (20.0%). Grade 3/4 anemia (20.0%) and dose decreases due to anemia (11.1%) or thrombocytopenia (6.7%) were infrequent.

**Conclusions:**

A dose-escalation approach may mitigate worsening anemia during early ruxolitinib therapy in some patients with myelofibrosis.

**Trial registration:**

ClinicalTrials.gov identifier, NCT01445769. Registered September 23, 2011.

## Background

Ruxolitinib, an orally bioavailable potent inhibitor of Janus kinase (JAK)1/JAK2 signaling, is approved for use in a twice-daily (BID) dosage regimen to treat intermediate- or high-risk myelofibrosis (MF) [1]. Dysregulation of the JAK signaling pathway is thought to play a central role in the pathobiology of MF, and 50 to 60% of patients with MF harbor a *JAK2* V617F gain-of-function mutation [2–4]. Ruxolitinib has been shown to inhibit proliferation of hematopoietic progenitor cells, improve splenomegaly, and prolong overall survival in preclinical models of disease [5], as well as in patients with MF [6–8]. In the phase 3 Controlled Myelofibrosis Study with Oral JAK Inhibitor Treatment (COMFORT)-I and COMFORT-II studies, administration of ruxolitinib reduced splenomegaly, as measured by magnetic resonance imaging [7, 8], and improved symptoms, as measured by the Myelofibrosis Symptom Assessment Form Total Symptom Score (MFSAF TSS) in COMFORT-I [7, 9] and the European Organization for Research and Treatment of Cancer Quality of Life questionnaire core model for global health status in COMFORT-II [8]. Although baseline hemoglobin levels were grade 0/1 for most patients [7, 8], grade 3/4 anemia was reported as an adverse event (AE) for 45.2 and 42% of patients receiving ruxolitinib, compared with 19.2 and 31% of those receiving placebo in COMFORT-I and best available therapy in COMFORT-II, respectively [7, 8]. Additionally, overall rates of thrombocytopenia were increased in patients receiving ruxolitinib compared with placebo in COMFORT-I (any grade, 70 vs 31%, respectively; grade 3/4, 13 vs 1%) [7] and with best available therapy in COMFORT-II (any grade, 68 vs 27%; grade 3/4, 8 vs 7%) [8]. High rates of anemia and thrombocytopenia within the initial weeks of ruxolitinib therapy may lead to discontinuation in clinical practice, and therefore subsequent risk of disease rebound.

Criteria for dose adjustments in the COMFORT studies focused on managing decreases in platelet and neutrophil counts and did not provide specific dose modification criteria for new-onset or continuing anemia. In COMFORT-I, approximately one half of all grade 3/4 anemia and grade 3/4 thrombocytopenia observed in patients receiving ruxolitinib occurred during the first 8 weeks of therapy [7, 10]. In both studies, mean hemoglobin reached a nadir after 8 to 12 weeks of ruxolitinib treatment, then recovered to new steady-state levels by week 24 [7, 8, 10]. Similarly, platelet counts primarily decreased in the first 8 to 12 weeks of ruxolitinib treatment before stabilizing over the longer term [10]. The dose adjustment approach used in the COMFORT studies resulted in low rates of discontinuation due to anemia or thrombocytopenia [7, 8, 10], but the proportion of patients receiving transfusions peaked between weeks 8 and 12 [7, 11].

Similar dose-escalation strategies to those described in the current study are being pursued in patients with MF with baseline/screening platelet counts of 50–100 × 10^9^/L in an ongoing phase 2 study in which ruxolitinib was administered at a starting dose of 5 mg BID [12], and an ongoing phase 1b study in which ruxolitinib was initiated at 5 mg BID, 5 mg AM/10 mg PM, 10 mg BID, 10 mg AM/15 mg PM, or 15 mg BID [13]. The present study explored whether a ruxolitinib dose-escalation strategy could provide clinically meaningful reductions in splenomegaly and symptoms while abating the early hematologic toxicities observed during the first 8 to 12 weeks of ruxolitinib therapy in patients with baseline platelet counts ≥ 100 × 10^9^/L. A regimen using a lower starting dose followed by incremental dose increases might decrease the frequency and severity of new-onset or worsening anemia or thrombocytopenia.

The current study evaluated an alternative dosing regimen (ruxolitinib 10 mg BID starting dose) in patients with primary MF (PMF), post-polycythemia vera MF (PPV-MF), or post-essential thrombocythemia MF (PET-MF). The effects of starting ruxolitinib at 10 mg BID on spleen volume, palpable spleen length, symptom burden, and safety and tolerability of ruxolitinib, including evaluation of new-onset transfusion dependence and grade ≥ 3 anemia, were assessed.

## Methods

### Study design

INCB 18424-261 (NCT01445769) was an open-label, multicenter, single-arm, phase 2 study evaluating an alternative dose regimen of ruxolitinib in adults with PMF, PET-MF, or PPV-MF. The study consisted of four phases: screening (up to 21 days), baseline (7 days), treatment (24 weeks), and follow-up (30–37 days after the last on-study ruxolitinib dose).

### Inclusion/exclusion criteria

All patients were men or women (age ≥ 18 years) diagnosed with PMF, PPV-MF, or PET-MF; platelet counts ≥ 100 × 10^9^/L; and hemoglobin levels ≥ 6.5 g/dL; all were willing to receive blood transfusions. At screening, all patients had a palpable spleen, life expectancy > 6 months, and an Eastern Cooperative Oncology Group (ECOG) performance status of 0 to 3. At screening and baseline, all patients had < 5% peripheral blasts. Prior MF therapies were permitted, but patients were required to discontinue all drugs used to treat underlying MF disease no later than day –1 of treatment initiation.

Patients were excluded if they had MF disease that was well controlled with current therapy per investigator assessment or had inadequate bone marrow reserves, demonstrated by absolute neutrophil count (ANC) < 1 × 10^9^/L or platelet count < 100 × 10^9^/L. Patients also were excluded if they had inadequate liver or renal function (direct bilirubin ≥ 2× the upper limit of normal [ULN], alanine aminotransferase > 2.5× ULN, or glomerular filtration rate < 30 mL/min), an invasive malignancy within the previous 5 years, recent severe or unstable cardiac disease, or an unknown transfusion history during the 12 weeks before screening.

### Dosage and administration of study drug

Ruxolitinib was administered orally as 5-mg tablets for 24 weeks. The initial ruxolitinib dose for all patients was 10 mg BID. Doses were taken in the morning and evening, approximately 12 h apart, without regard to food intake. Doses could be adjusted at any point during the study for safety, including protocol-defined anemia, declining platelet count, or declining ANC count (Table 1).

**Table 1:** Ruxolitinib dose modifications for safety

Patient status	Maximum ruxolitinib dose or action taken
Protocol-defined anemia*	5 mg BID^†^
Platelet count 75 to < 100 × 10^9^/L (inclusive)	10 mg BID
Platelet count 25 to < 75 × 10^9^/L	5 mg BID^‡^
Platelet count < 25 × 10^9^/L or ANC < 0.5 × 10^9^/L	Interrupt administration

*ANC* absolute neutrophil count, *BID* twice daily

*For patients who were transfusion-dependent at baseline, anemia was defined as a ≥ 50% increase in transfusion frequency vs the transfusion frequency before day 1. For patients who were transfusion-independent at baseline, anemia was defined as (1) a ≥ 2 g/dL decline in hemoglobin to < 8 g/dL, unless not confirmed by repeat laboratory assessment within 7 days without an intervening change in dose, use of an erythropoiesis stimulant, or receipt of a transfusion; or (2) receipt of any transfusions (2 units minimum) in the previous 6-week period

^†^If protocol-defined anemia occurred at a dose of 5 mg BID (after, for example, a dose reduction for declining platelet counts), the dose could continue at 5 mg BID

^‡^Patients already receiving 5 mg BID could continue at 5 mg BID with further declines in platelets to < 75 × 10^9^/L and ≥ 25 × 10^9^/L, but dosing was interrupted if platelet count was < 25 × 10^9^/L

With the exception of any safety-related dose interruptions or reductions, the 10-mg BID starting dose of ruxolitinib was maintained through week 12. At weeks 12 and 18, doses could be increased by 5 mg BID up to a maximum dose of 20 mg BID in patients with demonstrated lack of efficacy, defined as palpable spleen length below the costal margin reduced by < 40% from baseline or a change in Patient’s Global Impression of Change (PGIC) score from 3 to 7 [14]. The PGIC was used to evaluate patients’ overall sense of treatment effect on their MF symptoms using a 7-point scale: 1, very much improved; 2, much improved; 3, minimally improved; 4, no change; 5, minimally worse; 6, much worse; 7, very much worse. Additional criteria for dose increases at weeks 12 and 18 included protocol-defined minimum platelet count (week 12: ≥ 100 × 10^9^/L at study visit and ≥ 75 × 10^9^/L during the previous 6 weeks; week 18: ≥ 150 × 10^9^/L at study visit and ≥ 100 × 10^9^/L during the previous 6 weeks) and lack of any safety-related dose interruptions or reductions (Table 1). No dose increases were permitted beyond week 18. Patients benefiting from treatment were able to participate in an extension phase of the study.

The primary study endpoint was the mean percentage change from baseline in spleen volume at week 24 measured by independent central review of magnetic resonance imaging (MRI) or computed tomography scans in patients who were not candidates for MRI or if MRI was not readily available. Secondary endpoints included spleen measurements; MF symptoms, as measured by the modified Myelofibrosis Symptom Assessment Form v2.0 diary total symptom score (MFSAF TSS; sum of seven symptoms, including night sweats, itching, abdominal discomfort, pain under left ribs, early satiety, muscle/bone pain, and inactivity); transfusion status; and safety and tolerability. Spleen length was assessed by manual palpation using a soft ruler to measure from the left costal margin (typically at the midclavicular line) to the point of greatest splenic protrusion. Mean percentage change in palpable spleen length from baseline and the proportion of patients with ≥ 40% reduction in palpable spleen length were assessed. The 40% reduction represents a robust response, as a 25 to < 50% reduction in palpable spleen length in ruxolitinib-treated patients was associated with an approximate 35% reduction in spleen volume in a secondary analysis of the COMFORT-I trial [15]. Pre-specified safety assessments included evaluation of clinically notable anemia, defined as new onset of grade ≥ 3 anemia in patients who were transfusion-independent at baseline, new onset of transfusion dependence in patients who were transfusion-independent at baseline, or a 50% increase in transfusions from baseline in patients who were transfusion-dependent at baseline.

### Statistical analysis

The primary endpoint was analyzed using the intent-to-treat population (1-sample *t* test). The mean and median change and percentage change in spleen volume from baseline (weeks 12 and 24), the percentage of patients with ≥ 35% (based on the COMFORT-I and COMFORT-II primary endpoints [7, 8]), or ≥ 10% (arbitrary cut-off to identify small but durable changes in spleen volume) reduction in spleen volume from baseline at week 24, change in palpable spleen length from baseline to every study visit, and percentage change from baseline to every study visit were each estimated with 95% confidence intervals (CIs). The proportions of patients with a ≥ 50 or ≥ 20% reduction from baseline in MFSAF TSS (week 24) and the mean and median change and percentage change from baseline in individual symptoms and MFSAF TSS (weeks 6, 12, 18, and 24) were also estimated with 95% CIs.

Clinical safety data were analyzed using summary statistics. Descriptive statistics were provided for duration of treatment, average daily dose (mg) of ruxolitinib, and total dose (mg) of ruxolitinib for safety-evaluable patients. For key laboratory parameters, value was graded based on the Common Terminology Criteria for Adverse Events v4.03 grading system, and incidence rates of newly occurring or worsening abnormalities were calculated. Transfusion dependence was defined as receipt of ≥ 2 units of packed red blood cells in a ≤ 12-week interval.

Efficacy analyses were performed for the overall study population as well as on cohorts stratified by ruxolitinib final titrated daily dose. All tables, graphs, and statistical analyses were generated using SAS® software (SAS Institute Inc., Cary, NC; version 9 or later). All CIs were two-sided 95%, unadjusted for multiplicity.

## Results

### Patient disposition

Forty-five patients were enrolled in the study (Table 2). Forty-two patients (93.3%) completed the 24-week treatment period; five were lost to follow-up before the end of the study. The remaining 37 patients (82.2%) completed the study through the follow-up phase.

**Table 2:** Patient disposition

Disposition, *n* (%)	Patients (*N* = 45)
Completed through week 24	42 (93.3)
Discontinued during the treatment phase	3 (6.7)
Consent withdrawn	2 (4.4)
Disease progression	1 (2.2)
Deaths*	1 (2.2)
Completed the study^†^	37 (82.2)

*The patient who withdrew because of disease progression later died

^†^Completed the study, including the follow-up phase, which was up to 37 days after the final ruxolitinib dose. One patient was lost to follow-up and four patients discontinued during the study for other reasons

### Patient demographics and disease characteristics

The study population included 24 men and 21 women, with a median (range) age of 70 (48–85) years; 95.6% were white. Most patients (82.2%) had an ECOG performance status of 0 to 1, 68.9% had a Dynamic International Prognostic Scoring System (DIPSS) score of 1 to 2 (intermediate-1 disease risk), and 64.4% had *JAK2* mutations (Table 3). Per protocol, all patients had platelet counts ≥ 100 × 10^9^/L at study entry.

**Table 3:** Patient demographics and baseline disease characteristics*

Characteristic	Patients (*N* = 45)
Median (range) age, years	70 (48–85)
45–< 65, *n* (%)	8 (17.8)
65–< 75, *n* (%)	21 (46.7)
≥ 75, *n* (%)	16 (35.6)
Sex, *n* (%)	
Male	24 (53.3)
Female	21 (46.7)
Race, *n* (%)	
White	43 (95.6)
Black	1 (2.2)
Other	1 (2.2)
Median (range) height, cm	168 (152–193)
Median (range) weight, kg	74 (46–114)
Type of MF, *n* (%)	
PMF	25 (55.6)
PPV-MF	13 (28.9)
PET-MF	7 (15.6)
Median (range) spleen volume,^†^ cm^3^	1798.5 (763.2–6633.4)
Median (range) palpable spleen length below costal margin, cm	13 (0–34)
Prior hydroxyurea use, *n* (%)	29 (64.4)
ECOG performance status, *n* (%)	
0	17 (37.8)
1	20 (44.4)
2	7 (15.6)
3	1 (2.2)
DIPSS score, *n* (%)	
High risk (5–6)	6 (13.3)
Intermediate-2 (3–4)	7 (15.6)
Intermediate-1 (1–2)	31 (68.9)
Low (0)	1 (2.2)
Transfusion status, *n* (%)	
Independent	30 (66.7)
Dependent	15 (33.3)
*JAK2* mutation status, *n*^‡^ (%)	
Present	29 (64.4)
Absent	15 (33.3)
Median (range) V617F at baseline for patients with *JAK2* mutation, %^§^	77 (1–96)

*DIPSS* Dynamic International Prognostic Scoring System, *ECOG* Eastern Cooperative Oncology Group, *JAK* Janus kinase, *MF* myelofibrosis, *PET-MF* post-essential thrombocythemia myelofibrosis, *PMF* primary myelofibrosis, *PPV-MF* post-polycythemia vera myelofibrosis

*Intent-to-treat population

^†^*n* = 42

^‡^One patient had a missing baseline value for JAK mutation status

^§^*n* = 29

### Efficacy

#### Spleen volume

**Fig. 1:**
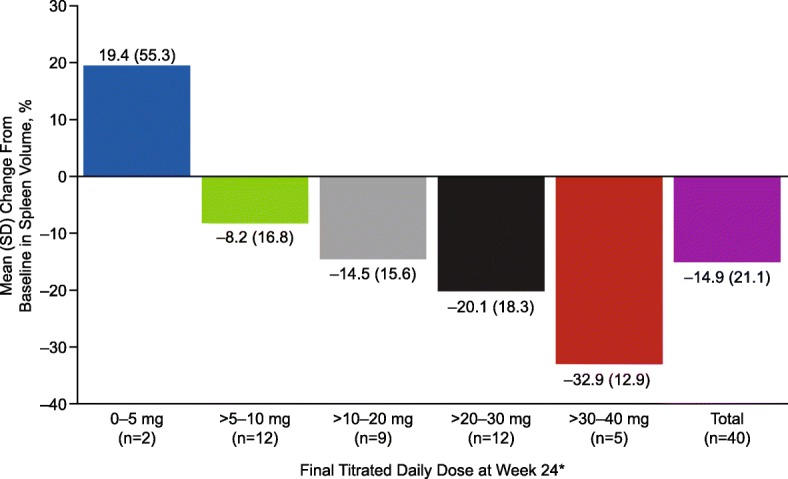
Mean percentage change in spleen volume from baseline to week 24. Includes patients from the intent-to-treat population with data at week 24. *The average daily dose during the 28 days before the spleen volume assessment (inclusive) at week 24

**Fig. 2:**
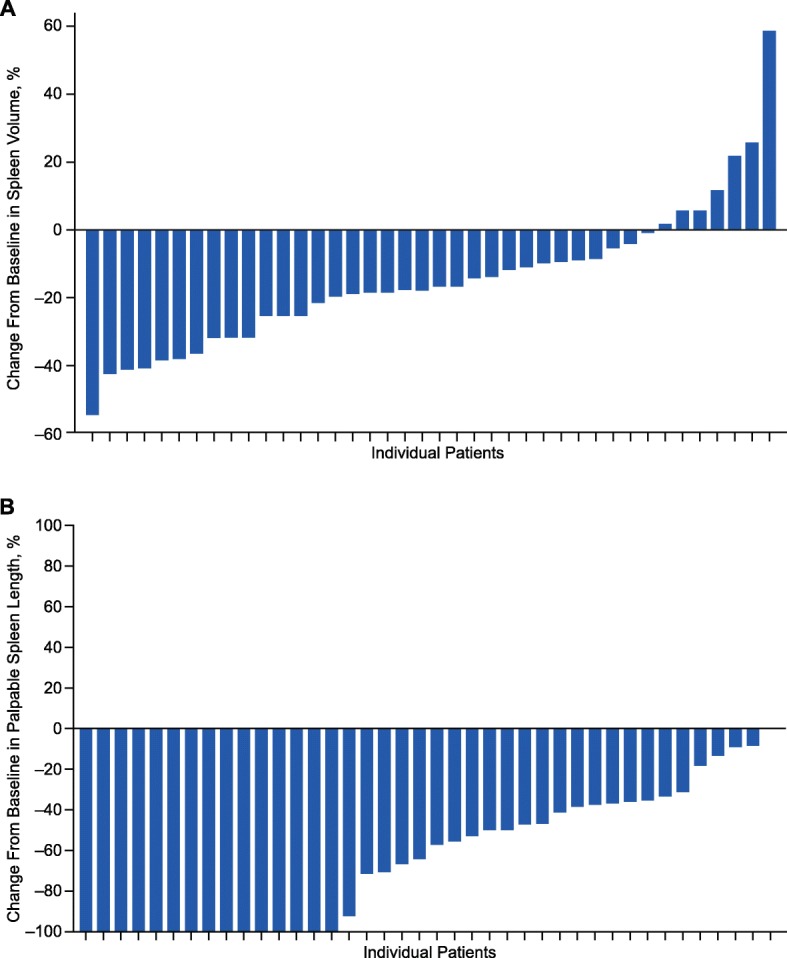
Maximum change in **a** spleen volume and **b** palpable spleen length from baseline to week 24. Includes patients from the intent-to-treat population (*n* = 40). Each bar represents an individual patient

The mean (standard deviation [SD]) percentage reduction from baseline in spleen volume at weeks 12 and 24 was 16.3% (12.4%) and 14.9% (21.1%), respectively. A clear dose-response relationship was observed, with week 24 mean (SD) spleen volume reductions of 20.1% (18.3%) and 32.9% (12.9%) among patients receiving average total daily doses of ruxolitinib > 20 to 30 mg and > 30 to 40 mg, respectively (Fig. 1). The median (range) change from baseline in spleen volume at week 24 was − 17.3% (− 54.2 to 58.5%; Fig. 2). Three patients had a > 20% increase in spleen volume from baseline, which influenced the mean value. At week 24, 26 patients (57.8%) had a ≥ 10% reduction in spleen volume from baseline; the week 24 total daily ruxolitinib dose distribution was as follows: > 0 to 5 mg, *n* = 1; > 5 to 10 mg, *n* = 6; > 10 to 20 mg, *n* = 6; > 20 to 30 mg, *n* = 8; > 30 to 40 mg, *n* = 5. At week 24, seven patients (15.6%) had ≥ 35% reduction in spleen volume (95% CI 4.97–26.14) from baseline; the week 24 total daily ruxolitinib dose distribution was as follows: > 5 to 10 mg, *n* = 1; > 20 to 30 mg, *n* = 3; > 30 to 40 mg, *n* = 3.

#### Other spleen measurements

The mean percentage reduction in palpable spleen length was approximately 40% from study week 6 to week 18. At week 24, the mean reduction in palpable spleen length from baseline was 47.6%. The proportions of patients with a ≥ 40% reduction from baseline in palpable spleen length at study weeks 6, 12, 18, and 24 were 44.4, 35.6, 31.1, and 44.4%, respectively.

#### MF symptoms

**Fig. 3:**
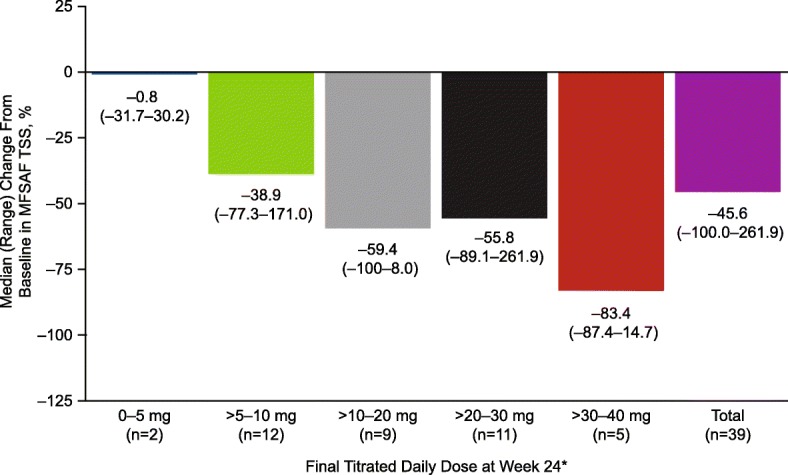
Median percentage change in MFSAF TSS from baseline to week 24. Includes patients from the intent-to-treat population with data at week 24. MFSAF TSS, Myelofibrosis Symptom Assessment Form Total Symptom Score. *The average daily dose during the 28 days before the spleen volume assessment (inclusive) at week 24

The mean (SD) MFSAF TSS at baseline and study weeks 6, 12, 18, and 24 were 16.6 (10.1), 10.5 (7.2), 10.0 (7.6), 8.4 (6.4), and 9.3 (8.0), respectively. The median (range) percentage changes in MFSAF TSS from baseline at study weeks 6, 12, 18, and 24 were − 31.3% (− 89.3 to 265.7%), − 39.3% (− 96.8 to 380.0%), − 49.3% (− 98.0 to 223.8%), and − 45.6% (− 100.0 to 261.9%), respectively. At week 24, patients receiving an average daily ruxolitinib dose > 30 to 40 mg had the greatest median percentage reduction from baseline in MFSAF TSS (Fig. 3).

At study weeks 12 and 24, 30 patients (66.7%) had a ≥ 20% reduction in MFSAF TSS from baseline. At week 24, MFSAF TSS reductions ≥ 20% were observed in 56.3% of patients who received ruxolitinib ≤ 10 mg daily and 72.4% of patients who received > 10 mg daily.

At weeks 12 and 24, 17 patients (37.8%) and 18 patients (40.0%), respectively, achieved a ≥ 50% improvement in MFSAF TSS from baseline. At week 24, ≥ 50% MFSAF TSS reductions were observed in 12.5% of patients who received an average daily ruxolitinib dose ≤ 10 mg and in 55.2% of patients who received an average daily ruxolitinib dose > 10 mg.

At week 24, ≥ 30% of patients had a ≥ 50% reduction of all individual components of the MFSAF TSS from baseline. At weeks 12 and 24, 19 patients (42.2%) and 20 patients (44.4%), respectively, had a ≥ 50% reduction in the abdominal symptom score (composite score for abdominal discomfort, pain under ribs on left side, and early satiety) from baseline.

### Safety

#### Exposure

All 45 patients received ≥ 1 dose of ruxolitinib. The median (range) duration of ruxolitinib treatment was 169 (31–257) days. The mean and median total daily doses of ruxolitinib were 19.7 and 20 mg, respectively, corresponding to approximately 10 mg BID.

#### Adverse events

Overall, 42 patients (93.3%) had a treatment-emergent AE (TEAE); anemia (26.7%) was the most frequently reported, followed by fatigue (22.2%) and arthralgia (20.0%). TEAEs occurring in ≥ 10% of patients and all grade 3/4 TEAEs are summarized in Table 4. Seventeen patients (37.8%) had a TEAE of grade 3 or higher, and 24 patients (53.3%) had treatment-related AEs. One TEAE (myelodysplastic syndrome [MDS]) led to discontinuation of ruxolitinib and study withdrawal for one patient. Serious AEs were reported in two patients (4.4%; cholelithiasis and dehydration occurring in one patient each), and the patient with MDS died of MDS during the study.

**Table 4:** TEAEs occurring in ≥ 10% of patients and any grade 3/4 TEAEs*

Preferred term, *n* (%)	Ruxolitinib (*N* = 45)	
	TEAEs	Grade 3/4 TEAEs
Any	42 (93.3)	17 (37.8)
Anemia	12 (26.7)	9 (20.0)
Fatigue	10 (22.2)	0
Arthralgia	9 (20.0)	0
Nausea	8 (17.8)	0
Thrombocytopenia	8 (17.8)	1 (2.2)
Dizziness	7 (15.6)	1 (2.2)
Abdominal pain	6 (13.3)	0
Cough	6 (13.3)	0
Diarrhea	6 (13.3)	0
Edema peripheral	6 (13.3)	0
Muscle spasms	6 (13.3)	0
Pain in extremity	6 (13.3)	0
Back pain	5 (11.1)	0
Contusion	5 (11.1)	0
Umbilical hernia	0	1 (2.2)
Cholelithiasis	0	1 (2.2)
Dehydration	0	1 (2.2)
Blood creatine phosphokinase increased	0	1 (2.2)
Blood triglycerides increased	0	1 (2.2)
Lipase increased	0	1 (2.2)
Hyperkalemia	0	1 (2.2)
Hypermagnesemia	0	1 (2.2)
Myelodysplastic syndrome^**†**^	0	1 (2.2)

*TEAE* treatment-emergent adverse event

*Safety-evaluable population

^†^Myelodysplastic syndrome was the only grade 4 TEAE

Nine patients (20.0%) had TEAEs leading to dose reduction. The most frequently reported TEAEs leading to dose decrease were anemia (*n* = 5, 11.1%) and thrombocytopenia (*n* = 3, 6.7%).

#### Hematologic parameters

**Fig. 4:**
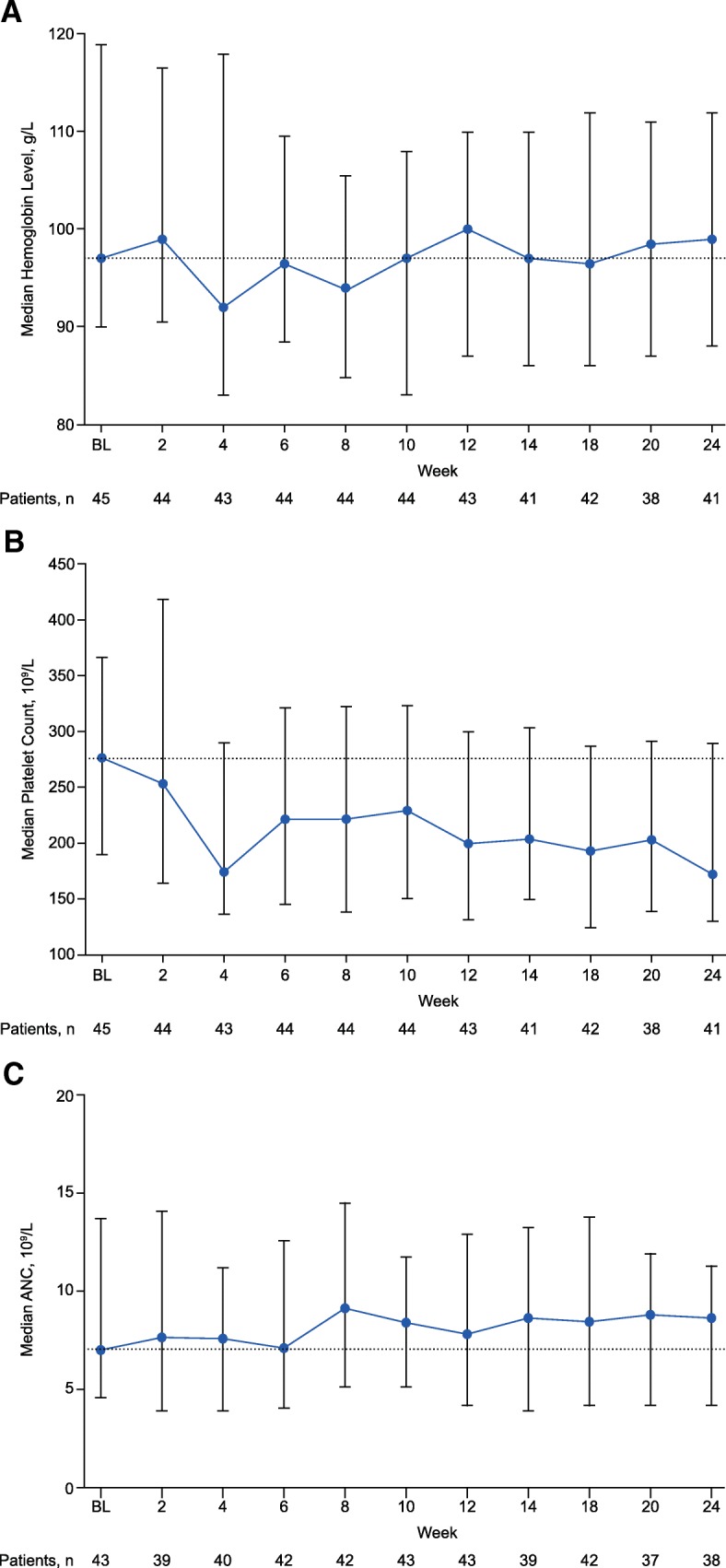
Hematologic parameters: **a** median hemoglobin levels, **b** platelet counts, and **c** ANCs over time. Includes patients from the safety-evaluable population. Error bars represent 25th and 75th percentiles. Dotted lines represent the median value at baseline. ANC, absolute neutrophil count; BL, baseline

Figure 4 depicts median hemoglobin, platelets, and ANC through study week 24. After a small decrease at week 4, median hemoglobin returned to baseline levels at week 10 and remained similar to baseline through week 24. Twenty patients (44.4%) had a grade 3 decrease in hemoglobin level on study; no patients had a grade 4 decrease.

Median platelet count declined from baseline (277 × 10^9^/L) in the first 4 weeks of treatment to 176 × 10^9^/L, then increased and remained in a range of 193.5 × 10^9^/L to 230.0 × 10^9^/L from week 6 to week 20 (median 172 × 10^9^/L at week 24). One patient each had a grade 3 and grade 4 thrombocytopenia on study. In general, median ANC counts remained stable through week 24.

#### Transfusions

Of the 30 patients who were red blood cell transfusion-independent at baseline, 21 (70.0%) maintained transfusion independence throughout the treatment phase of the study. Of the 15 patients who were red blood cell transfusion-dependent at baseline, a shift to transfusion independence occurred in one patient (6.7%) from day 1 to week 12 and in three patients each (20.0%) during weeks 6 to 18 and weeks 12 to 24.

## Discussion

This 24-week, open-label study examined whether initiating ruxolitinib therapy at a lower dose affected the initial drop in hemoglobin that was observed during the first 8 to 12 weeks of therapy in COMFORT-I [7] and COMFORT-II [8], while retaining efficacy. This study protocol allowed clinicians the opportunity to titrate doses based on safety and efficacy and resulted in lower rates of grade 3/4 anemia compared with COMFORT-I and COMFORT-II (grade 3: 20.0% vs 34% and 34%, respectively; grade 4: 0% vs 11% and 8%) [1, 8]. Furthermore, the majority of patients who were transfusion-independent at baseline remained so during the study. Although the patient population for this study was small, with 37 patients (82% of initial enrollment) completing through the follow-up phase, these findings suggest that a dose-escalation approach may be advantageous in patients for whom anemia is, or is likely to become, a problem while receiving ruxolitinib therapy (i.e., patients with low baseline hemoglobin levels).

Eligibility criteria and patient populations in this study and in COMFORT-I [7] and COMFORT-II [8] were similar. However, unlike the COMFORT-I and COMFORT-II studies, in which nearly all patients (> 99%) treated with ruxolitinib were intermediate-2 or high-risk as assessed by the International Prognostic Scoring System [7, 8], more than two thirds (68.9%) of patients in the current study were intermediate-1 risk status at baseline, and 28.9% had DIPSS scores indicating intermediate-2 or high-risk. Current National Comprehensive Cancer Network (NCCN) clinical practice guidelines recommend treatment with ruxolitinib for patients with intermediate-1, intermediate-2, and high-risk MF, depending on their symptom status (intermediate-1 MF) or their transplant eligibility and platelet status (intermediate-2 or high-risk MF) [16]. Per these NCCN guidelines and based on the presence of symptomatic splenomegaly and/or constitutional symptoms, intermediate-1 risk patients enrolled in this study had indications for ruxolitinib treatment similar to those of intermediate-2 risk and high-risk patients.

In the present study, reduction in spleen volume from baseline showed a clear dose response. Mean week 24 reductions in spleen volume in COMFORT-I and COMFORT-II were 31.6 and 29.2%, respectively, among patients taking initial ruxolitinib doses of 15 or 20 mg BID (decreased to 10–15 mg BID during the first 8–12 weeks in some cases) [7, 8, 10]. Interestingly, compared with the COMFORT studies [8, 10], spleen volume decreased less at week 24 across all dose levels in the present study. It should also be noted that 58.7 and 32% of patients originally randomized to ruxolitinib in COMFORT-I and COMFORT-II, respectively, achieved a ≥ 35% reduction in spleen volume during study follow-up, most by week 12, as compared with 15.6% in the present study. The correlation between reductions in palpable spleen length and spleen volume was also higher in COMFORT-I than in the present study [8, 17].

Compared with changes in spleen volume, changes in MFSAF TSS depended less on ruxolitinib dose. The median percentage reduction (improvement) in MFSAF TSS was 45.6% at 24 weeks. This finding was similar to 24-week data from COMFORT-I, in which median reduction in MFSAF TSS was 56.2% [7]. Notably, in COMFORT-I, the majority of patients with a ≥ 50% reduction in MFSAF TSS characterized their disease as “improved” or “very much improved” per PGIC [18].

The alternative dosing scheme explored in this study (ruxolitinib 10 mg BID) provided benefit with regard to symptoms and reduced palpable spleen length in most patients; however, improvements in efficacy outcomes observed here with the 10-mg BID dose were smaller compared with those observed in COMFORT-I, in which the mean doses at the end of 24 weeks were ~ 10 mg (for a starting dose of 15 mg BID) and 15 to 20 mg BID (for a starting dose of 20 mg BID) [10]. In COMFORT-II, starting doses were 15 mg BID in 38% of patients and 20 mg BID in 62% of patients (median [range] dose intensity of ruxolitinib, 30 [10–49] mg/day). The difference may have occurred because of the protocol’s mandate to increase the dose of study drug only for patients who had a reduction in palpable spleen length < 40% (or a PGIC of minimally improved or worse), preventing patients from attaining greater reductions in spleen volume with higher doses.

Current guidelines for the use of ruxolitinib to manage symptoms consider dose-response relationships and dose adjustments in the development of specific recommendations for the clinical management of MF. The European LeukemiaNet and the Italian Society of Hematology have strongly recommended ruxolitinib for improving symptomatic or severe splenomegaly (> 15 cm below the costal margin) in patients with intermediate-2 or high-risk disease, as well as for improving systemic symptoms in patients with a score of ≥ 44 on the MPN10 (a validated tool used to assess the severity of 10 symptoms related to myeloproliferative neoplasms), refractory severe itching, unintended weight loss not attributable to other causes, or unexplained fever [19]. However, the panel provided no specific recommendations regarding the tapering of drug doses, the use of combination therapies, or the method and timing of response assessment.

## Conclusions

Concerns of progressive cytopenias in patients with MF treated with ruxolitinib are well established. Results from this open-label, multicenter, single-arm, phase 2 study suggest that initiating therapy at lower doses can be performed safely and may provide clinical benefit, including improvements in splenomegaly and symptoms, in patients with MF for whom anemia is, or is likely to become, a concern while receiving treatment with ruxolitinib. Current recommendations, including those detailed in the NCCN Clinical Practice Guidelines in Oncology: Myeloproliferative Neoplasms [16], continue to adhere to ruxolitinib dosing as described in the product label [1], in which the initial ruxolitinib dose is based on baseline platelet count. However, the NCCN does recognize that there are specific clinical situations that may support the initiation of ruxolitinib at a lower dose, followed by dose increases [16]. Alternative strategies should be considered for patients who have anemia or are likely to become anemic.

## Abbreviations

AE: Adverse event
ANC: Absolute neutrophil count
BID: Twice daily
CI: Confidence interval
COMFORT: Controlled Myelofibrosis Study with Oral JAK Inhibitor Treatment
DIPSS: Dynamic International Prognostic Scoring System
ECOG: Eastern Cooperative Oncology Group
GCP: Good Clinical Practice
JAK: Janus kinase
MDS: Myelodysplastic syndrome
MF: Myelofibrosis
MFSAF TSS: Myelofibrosis Symptom Assessment Form Total Symptom Score
NCCN: National Comprehensive Cancer Network
PET-MF: Post-essential thrombocythemia myelofibrosis
PGIC: Patient’s Global Impression of Change
PMF: Primary myelofibrosis
PPV-MF: Post-polycythemia vera myelofibrosis
SD: Standard deviation
TEAE: Treatment-emergent adverse event
ULN: Upper limit of normal

## References

[1] JAKAFI® (ruxolitinib) (2017). Full prescribing information.

[2] Agarwal A, Morrone K, Bartenstein M, Zhao ZJ, Verma A, Goel S (2016). Bone marrow fibrosis in primary myelofibrosis: pathogenic mechanisms and the role of TGF-beta. Stem Cell Investig.

[3] Baxter EJ, Scott LM, Campbell PJ, East C, Fourouclas N, Swanton S (2005). Acquired mutation of the tyrosine kinase JAK2 in human myeloproliferative disorders. Lancet.

[4] Kralovics R, Passamonti F, Buser AS, Teo SS, Tiedt R, Passweg JR (2005). A gain-of-function mutation of JAK2 in myeloproliferative disorders. N Engl J Med.

[5] Quintás-Cardama A, Vaddi K, Liu P, Manshouri T, Li J, Scherle PA (2010). Preclinical characterization of the selective JAK1/2 inhibitor INCB018424: therapeutic implications for the treatment of myeloproliferative neoplasms. Blood.

[6] Verstovsek S, Kantarjian H, Mesa RA, Pardanani AD, Cortes-Franco J, Thomas DA (2010). Safety and efficacy of INCB018424, a JAK1 and JAK2 inhibitor, in myelofibrosis. N Engl J Med.

[7] Verstovsek S, Mesa RA, Gotlib J, Levy RS, Gupta V, DiPersio JF (2012). A double-blind, placebo-controlled trial of ruxolitinib for myelofibrosis. N Engl J Med.

[8] Harrison C, Kiladjian JJ, Al-Ali HK, Gisslinger H, Waltzman R, Stalbovskaya V (2012). JAK inhibition with ruxolitinib versus best available therapy for myelofibrosis. N Engl J Med.

[9] Mesa RA, Schwager S, Radia D, Cheville A, Hussein K, Niblack J (2009). The Myelofibrosis Symptom Assessment Form (MFSAF): an evidence-based brief inventory to measure quality of life and symptomatic response to treatment in myelofibrosis. Leuk Res.

[10] Verstovsek S, Mesa RA, Gotlib J, Levy RS, Gupta V, DiPersio JF (2013). Efficacy, safety and survival with ruxolitinib in patients with myelofibrosis: results of a median 2-year follow-up of COMFORT-I. Haematologica.

[11] Cervantes F, Vannucchi AM, Kiladjian JJ, Al-Ali HK, Sirulnik A, Stalbovskaya V (2013). Three-year efficacy, safety, and survival findings from COMFORT-II, a phase 3 study comparing ruxolitinib with best available therapy for myelofibrosis. Blood.

[12] Talpaz M, Paquette R, Afrin L, Hamburg SI, Prchal JT, Jamieson K (2013). Interim analysis of safety and efficacy of ruxolitinib in patients with myelofibrosis and low platelet counts. J Hematol Oncol.

[13] Vannucchi AM, Gisslinger H, Harrison CN, Al-Ali HK, Pungolino E, Kiladjian JJ (2015). EXPAND: a phase 1b, open-label, dose-finding study of ruxolitinib in patients with myelofibrosis (MF) and low platelet counts (50 × 10^9^/L to 99 × 10^9^/L) at baseline. Blood.

[14] Hurst H, Bolton J (2004). Assessing the clinical significance of change scores recorded on subjective outcome measures. J Manip Physiol Ther.

[15] Miller CB, Komrokji RS, Mesa RA, Sun W, Montgomery M, Verstovsek S (2017). Practical measures of clinical benefit with ruxolitinib therapy: an exploratory analysis of COMFORT-I. Clin Lymphoma Myeloma Leuk.

[16] National Comprehensive Cancer Network. NCCN clinical practice guidelines in oncology. Myeloproliferative neoplasms. Version 2.2018.Available at: https://www.nccn.org/professionals/physician_gls/pdf/mpn.pdf. Accessed 14 February 2018. 10.6004/jnccn.2016.0169 PMC11807340 27956542

[17] Verstovsek S, Mesa RA, Gotlib J, Levy RS, Gupta V, DiPersio JF (2015). Efficacy, safety, and survival with ruxolitinib in patients with myelofibrosis: results of a median 3-year follow-up of COMFORT-I. Haematologica.

[18] Mesa RA, Gotlib J, Gupta V, Catalano JV, Deininger MW, Shields AL (2013). Effect of ruxolitinib therapy on myelofibrosis-related symptoms and other patient-reported outcomes in COMFORT-I: a randomized, double-blind, placebo-controlled trial. J Clin Oncol.

[19] Marchetti M, Barosi G, Cervantes F, Birgegard G, Griesshammer M, Harrison C (2017). Which patients with myelofibrosis should receive ruxolitinib therapy? ELN-SIE evidence-based recommendations. Leukemia.

